# Efficiency evaluation and promoter identification of primary health care system in China: an enhanced DEA-Tobit approach

**DOI:** 10.1186/s12913-024-11244-0

**Published:** 2024-07-03

**Authors:** Zhi Zeng, Xiru Yu, Wenjuan Tao, Wei Feng, Wei Zhang

**Affiliations:** 1 Institute of Hospital Management, West China Hospital, Sichuan University, Chengdu, Sichuan 610041 China; 2 Office of Policy Research, Chinese Center for Disease Control and Prevention & Chinese Academy of Preventive Medicine, Beijing, China; 3 Institute for Hospital Management, Tsinghua University, Shenzhen, Guangdong 518055 China; 4 West China School of Public Health, West China Fourth Hospital, Sichuan University, Chengdu, Sichuan 610041 China; 5 West China Biomedical Big Data Center, West China Hospital, Sichuan University, Chengdu, Sichuan 610041 China; 6 Med-X Center for Informatics, Sichuan University, Chengdu, Sichuan 610041 China

**Keywords:** Primary health care, Efficiency evaluation, Horizontal integration, Data envelopment analysis, Tobit regression, China

## Abstract

**Background:**

With Primary Health Care (PHC) being a cornerstone of accessible, affordable, and effective healthcare worldwide, its efficiency, especially in developing countries like China, is crucial for achieving Universal Health Coverage (UHC). This study evaluates the efficiency of PHC systems in a southwest China municipality post-healthcare reform, identifying factors influencing efficiency and proposing strategies for improvement.

**Methods:**

Utilising a 10-year provincial panel dataset, this study employs an enhanced Data Envelopment Analysis (DEA) model integrating Slack-Based Measure (SBM) and Directional Distance Function (DDF) with the Global Malmquist-Luenberger (GML) index for efficiency evaluation. Tobit regression analysis identifies efficiency determinants within the context of China’s healthcare reforms, focusing on horizontal integration, fiscal spending, urbanisation rates, and workforce optimisation.

**Results:**

The study reveals a slight decline in PHC system efficiency across the municipality from 2009 to 2018. However, the highest-performing county achieved a 2.36% increase in Total Factor Productivity (TFP), demonstrating the potential of horizontal integration reforms and strategic fiscal investments in enhancing PHC efficiency. However, an increase in nurse density per 1,000 population negatively correlated with efficiency, indicating the need for a balanced approach to workforce expansion.

**Conclusions:**

Horizontal integration reforms, along with targeted fiscal inputs and urbanisation, are key to improving PHC efficiency in underdeveloped regions. The study underscores the importance of optimising workforce allocation and skillsets over mere expansion, providing valuable insights for policymakers aiming to strengthen PHC systems toward achieving UHC in China and similar contexts.

**Supplementary Information:**

The online version contains supplementary material available at 10.1186/s12913-024-11244-0.

## Background

Primary Health Care (PHC) systems are fundamental to health service delivery across the globe, serving as first point of contact for individuals within the healthcare spectrum. They are central to high-quality health systems, and responsible for providing essential medical and public health services [1]. Although countries have long stated their commitment to improving PHC, approximately 75% of the global population, including at least 85% of individuals in low- and middle-income countries, remain without access to accessible, affordable, and effective PHC [2]. The COVID-19 pandemic has brought the need for well-functioning PHC into sharp focus [3]. Despite the constraints on government investment, identifying strategies to expedite the development of PHC institutions and enhance their service efficiency within the existing investment framework emerges as a critical challenge in achieving Universal Health Coverage (UHC) [4]. In China, the PHC system is crucial in supporting the country’s vast population, particularly in rural and underserved areas. This system encompasses a wide array of facilities, including community health centres, township health centres, and village clinics, which together strive to deliver comprehensive, accessible, and culturally competent care to the community. These facilities are tasked with a broad range of functions from preventive care, treatment of common illnesses, and management of chronic conditions to maternal and child health services [5–7]. Despite their foundational role, Chinese PHC systems face numerous challenges that compromise their efficiency and effectiveness. These include issues such as inadequate funding, disparities in resource distribution, and shortages of qualified healthcare professionals [5, 6, 8–10]. Additionally, the rapid urbanisation and aging population bring about increased healthcare demands that further strain these resources [7, 11]. Given these challenges, the PHC system should be accorded higher priority in regional health affairs, with efforts directed towards transforming the existing fragmented and treatment-focused delivery system into a model emphasizing population health via high-quality, PHC-based integrated delivery [3, 7, 12, 13].

Evaluating the efficiency of the PHC system is crucial for optimising the allocation of limited resources and enhancing the sustainability of healthcare systems in the face of increasing healthcare demands. This study aims to assess the efficiency of PHC institutions in China and identify the key environmental factors influencing their efficiency, using robust methodologies to inform policy adjustments and resource allocation strategies.

In the following sections, we first review the existing literature on healthcare efficiency evaluation and the application of Data Envelopment Analysis (DEA) and Tobit regression in this context. We then describe our study design, including the selection of input and output variables for the DEA model, and the environmental factors considered in the Tobit regression. Finally, we present the results of our analysis and discuss their implications for policy and practice.

## Literature review

The Chinese PHC systems have been a critical area of research, particularly in the context of ongoing reforms aimed at enhancing service delivery and optimising resource allocation [5, 7]. The literature on this subject is extensive and illustrates a variety of methodologies employed to assess the efficiency of healthcare operations.

In this context, healthcare efficiency is defined as the maximization of health outcomes with available resources or the minimization of resources utilised to attain specified health outcomes. The evaluation of healthcare efficiency in China has garnered significant attention due to the country’s extensive healthcare reform initiatives. Studies such as those by Zhou et al. [4] and Hou et al. [14] have utilised traditional DEA to assess the performance of healthcare institutions across various provinces. These studies provide a foundational understanding of the areas where efficiency can be improved and highlight the impact of regional disparities on healthcare delivery.

Efficiency measurement in healthcare commonly employs non-parametric methods like DEA and parametric methods such as Stochastic Frontier Analysis (SFA) [15]. DEA is favored due to its flexibility in handling multiple inputs and outputs without the need for predefined functional forms [16]. Since its adoption in the mid-1980s, DEA has been extensively utilised to assess healthcare efficiency globally [17–19]. Despite its widespread use, traditional DEA models like Charnes-Cooper-Rhodes (CCR) and Banker-Charnes-Cooper (BCC) models often overlook the quality of outputs and the external environmental factors impacting institutional efficiency. Recognizing the limitations of traditional DEA, researchers have developed improved models such as the three-stage DEA approach [20]. The conventional DEA techniques have limitations in handling slack variables, while the traditional directional distance function (DDF) tends to introduce bias when slack variables exist [21]. In contrast, the Slack-Based Measure (SBM) has been shown to effectively overcome issues of slackness, providing a more robust analysis [22]. Enhancements continue with the integration of the non-radial, non-angular SBM model with DDF, which refines the accuracy of efficiency measurements significantly [23].

Tobit regression analysis has been effectively used to delve deeper into the determinants of efficiency. By handling censored data typical in DEA scores (where efficiency is bounded between 0 and 1), Tobit models allow researchers to estimate the impact of non-discretionary factors on efficiency levels. Studies by Banker and Natarajan have demonstrated the utility of this approach in adjusting DEA scores for external influences, thus providing a more nuanced analysis of efficiency determinants [24]. Studies Integrating DEA and Tobit Analysis have become increasingly popular for examining efficiency in the healthcare sector. This combined approach has been shown to provide valuable insights into how different factors, including policy changes and operational practices, influence the efficiency of healthcare institutions. For instance, research by Guo et al. used the Malmquist DEA model to measure efficiency and applied the Tobit regression to explore factors that influence the efficiency of government healthcare expenditure [25].

In terms of broader methodological applications, the Global Malmquist-Luenberger (GML) index has been increasingly adopted to capture dynamic changes in productivity, especially in the wake of substantial organizational reforms. The work of Oh stands as a seminal reference in this area, providing a robust framework for incorporating undesirable outputs in productivity measurement, which is crucial for a holistic assessment of healthcare reforms [26].

By synthesizing these diverse perspectives, our study stands on a robust foundation of empirical evidence and theoretical rigor, substantially enriching the dialogue on healthcare efficiency and policy development within the Chinese context. Utilising advanced analytical techniques, this study delves into the intricacies of the Chinese PHC system, making a significant contribution to the ongoing discussions about healthcare efficiency and policy development in China.

## Methods

### Study setting

Chongqing, one of China’s four municipalities directly governed by the central government, serves as an ideal setting for this study due to its distinctive administrative and economic autonomy. With a vast territory of 82,400 square kilometers and a population of 32.13 million across 38 diverse districts and counties, Chongqing represents a microcosm of the socioeconomic and healthcare challenges prevalent in central and western China [27]. The municipality has long faced prominent issues in its PHC system, such as difficulties in accessing medical services and high medical expenses, particularly in rural areas. By measuring changes in these undesirable output indicators, this study aims to evaluate the effectiveness of healthcare reform policies in alleviating these challenges. Given Chongqing’s unique characteristics, which are representative of many less-developed regions in China, the findings can offer valuable insights for improving PHC reform policies and measures at a broader scale. Thus, selecting Chongqing as the study area allows for an in-depth exploration of healthcare reforms’ impacts on PHC efficiency in settings marked by rapid urbanisation and significant rural healthcare delivery issues, providing lessons on the adaptability and scalability of healthcare reforms both within China and globally. Refer to Figure S1 in Additional File 1 for the Geographical representation of Chongqing.

### Data source

Statistics of PHC institutions across 38 counties and districts in the study municipality were derived from the *Chongqing Health Statistical Yearbook, Chongqing Health and Family Planning Statistical Yearbook*, and Statistical Bulletins from 2009 to 2018. The resulting balanced panel dataset empowers this research to evaluate the dynamic efficiency of the PHC system before and after the implementation of the latest profound health reform.

### Study design

This study is designed as a quantitative, longitudinal analysis utilising a robust 10-year provincial panel dataset spanning from 2009 to 2018. The start year, 2009, marks the initiation of China’s most recent major health reform, providing a unique pre-post-reform data environment. The endpoint of 2018 was strategically chosen because it precedes the implementation of new rounds of PHC reconstruction projects across several counties and districts, ensuring that the data reflects the outcomes of the aforementioned health reform without additional influences.

The analysis is meticulously structured around three critical steps:

- Enhanced DEA Model: The study evaluates the dynamic efficiency of PHC across all 38 districts/counties using an enhanced DEA model that incorporates the SBM and DDF. This model is specifically designed to overcome traditional DEA limitations, such as handling slack variables and biases. It effectively measures efficiency by considering both the radial and non-radial slacks, thereby enhancing the accuracy and reliability of the efficiency assessments.
- Calculation of the GML Index: Following the DEA, the study calculates the GML index to measure productivity changes over time. This index is critical as it accounts for undesirable outputs and productivity variations, offering a more nuanced measure of efficiency adapted to the complexities of healthcare. The GML index is particularly valuable for assessing the impact of healthcare reforms on PHC systems, providing insights into temporal efficiency trends, and highlighting areas for potential improvement.
- Tobit Regression Analysis: After establishing efficiency scores and productivity changes, Tobit regression analysis is conducted to delve deeper into the determinants of these efficiency changes. This model is adept at handling the censored nature of DEA efficiency scores and provides a robust mechanism to quantify how various external factors, such as fiscal policies, urbanisation rates, and workforce allocations, influence PHC efficiency.

### Indicators and variables

The indicators chosen for the DEA reflect core components of PHC efficiency, based on the Primary Health Care Performance Initiative (PHCPI) framework which is adapted to the specifics of the Chinese healthcare context. These indicators include a mix of inputs (such as the number of healthcare workers, facility count, and equipment levels) and outputs (such as patient satisfaction rates and treatment success rates). Undesirable outputs, crucial for understanding aspects of UHC such as accessibility and affordability issues, are also included to provide a comprehensive view of PHC performance.

#### Indicators for evaluation

To measure the efficiency of PHC, we started with the PHCPI framework which elucidates essential PHC components [28]. While challenges of difficulty in seeking medical services and high expenses pertaining to medical service utilisation reflect the UHC goals of essential health services coverage and financial risk protection to some extent, they also delineate features unique to the Chinese health system. Hence, a customized framework attuned to the Chinese setting served as guidance in the indicator selection process [13].

Common PHC efficiency evaluation indicators encompass institutions, beds, professionals, and equipment, while outputs normally include service utilisation metrics like visits and admissions alongside certain public health indicators [13, 14, 16, 20, 29–34]. However, few studies have incorporated undesirable outputs, which carry important information related to UHC dimensions tailored to the Chinese settings. In China’s healthcare context, undesirable outputs like excessively high or rapidly increasing per-inpatient expenses reflect challenges in achieving UHC goals of accessible and affordable essential health services for all and are thus considered undesirable outputs of the PHC system. Incorporating these measures into the evaluation framework allows a comprehensive assessment of PHC institutions’ efficiency and effectiveness in delivering quality, accessible, and affordable basic health services.

Ultimately, three key inputs, two desirable outputs capturing PHC service volume and breadth, and four undesirable outputs reflecting accessibility and affordability issues under UHC were selected, as exhibited in Table 1. To mitigate analytical biases by combining economic and quantitative markers, this study did not directly use economic indicators like fiscal input and revenue as indicators. Considering data availability, public health service indicators were not included as most literature did. The DEA evaluation method requires the number of units to exceed the number of evaluation indicators by at least twice [35].

**Table 1:** Description of selected indicators

Category	Variable	Description
Input	Number of physicians	Healthcare professionals embody PHC human resources investment
	Number of nurses	
	Number of available beds	Beds represent capital inputs for infrastructure and equipment
Desirable output	Outpatient visits*	Outpatient visits and discharged patients signify service utilization for disease treatment
	Inpatient admission	
Undesirable output	Proportion of outpatient* services at PHC institutions	Share of PHC services indicates convenience and trust for residents to choose PHC providers as first-contact care
	Proportion of inpatient services at PHC institutions	
	Per outpatient expenses	Excessively high or rapidly increasing per capita medical expenses can affect the financial affordability of patients
	Per inpatient expenses	

Note: * The total of outpatient visits and emergency visits

#### Variables for tobit regression model

Variations in uncontrollable factors spanning economic, social, and policy areas may also influence PHC efficiency. Hence, eleven variables on aspects of fiscal input, workforce allocation, material input, and socioeconomic context were chosen as preliminary explanatory predictors for panel regression, as exhibited in Table 2. Total factor productivity (TFP) as measured by the GML index was the key dependent variable, which signifies overall productivity changes, providing a robust measure incorporating inputs, desirable outputs, and undesirable outputs (Table 2).

**Table 2:** Definition of Tobit regression variables

Category	Variable	Definition
GML index	Y	Dependent variable
Fiscal input	X_1_	Proportion of fiscal expenses on PHC institutions (%)
	X_2_	Per capita fiscal expenses on PHC institutions (CNY)
	X_3_	Total revenue of PHC institutions (CNY)
Workforce	X_4_	Number of physicians per 1,000 resident population
	X_5_	Number of nurses per 1,000 resident population
Material input	X_6_	Number of beds per 1,000 resident population
	X_7_	Value of equipment above 10,000 CNY (10,000 CNY)
Socioeconomic factors	X_8_	Resident population of district/county (10,000 people)
	X_9_	Urbanization rate of district/county (%)
	X_10_	Per capita GDP of district/county (CNY)
	X_11_	Region: 1 = Metropolitan circle; 2 = Northeast urban cluster; 3 = Southeast urban cluster

Although education is a crucial determinant of workforce quality and operational efficiency, it was not included in the Tobit regression analysis due to the lack of comprehensive and consistent data on the education levels of healthcare staff across all surveyed PHC institutions.

Variable selection was informed by expert consultations and references to authoritative frameworks like PHCPI.

### Analysis

DEA enables the assessment of the relative efficiency of decision-making units (DMUs) without specifying functional forms [36]. DEA applied in efficiency analysis of the health industry showed ideal performance, particularly in multi-input multi-output scenarios [35]. Conventional DEA techniques like CCR and BCC have shortcomings in handling slack variables, and traditional DDF tends to introduce estimation bias when slack variables exist. Since the SBM model outperforms traditional ones when dealing with slackness issues [21], integrating the non-radial, non-angular SBM with DDF better reflects efficiency [23]. The customized SBM-DDF model provides methodological refinements tailored to the study’s context.

Referring to established literature, the Malmquist Luenberger index incorporating DDF can accommodate undesirable outputs [26]. A value exceeding 1 indicates an increase in TFP, a value below 1 signifies a decline, otherwise a stable state. The GML index represents the change from one period to the next, furnishing a robust fit for inputs, desirable outputs, and undesirable outputs.

A Tobit regression model was chosen to analyse the influencing factors of TFP and examine correlations [37]. Utilising TFP to reflect PHC service efficiency has rich research precedents [29, 34, 38, 39]. Its maximum likelihood estimation approach is well-suited for modelling the DEA efficiency scores as censored or truncated dependent variables. Though some studies noted the superiority of bootstrapping truncated regression [14], the Tobit model was more appropriate for directly modelling GML scores in this study with a relatively small sample size unfavorable for bootstrap resampling. Moreover, given the research aim of evaluating correlations, the Tobit model’s provision of parameter estimates and significance testing better met analytical needs.

Correlation analysis, multicollinearity tests, Hausman tests, and Likelihood Ratio (LR) were executed. If no multicollinearity is detected, and the p-value in the Hausman test and LR test are above the significance level (set at 5%), then the mixed Tobit model is proper.

In our analysis, we computed the GML index using MATLAB (Version R2018a, MathWorks, Inc., 2018). Tobit model regression results calculation and statistical tests were conducted with Stata16 software (StataCorporation, 2016). The key formulas and equations underpinning the methodological approaches are presented in the “Key Formulas and Equations” section of Additional File 1.

## Results

### Descriptive data

The study includes 38 evaluation units with a model comprising three input and six output indicators (two desirable and four undesirable). The sample size meets the model requirements. The descriptive statistics of relevant indicators are presented in Table S1, Additional File 1.

### GML results

As shown in Table 3, GML varied across all districts and counties, and the average value is 0.984, indicating a slight decrease (1.6%) of the TFP of PHC institutions in the research municipality. In contrast, the top-ranked county achieved the highest GML at 1.0236, suggesting a 2.36% increase in TFP for its PHC institutions in the 10-year period. GML breakdown indices are detailed in Table S2, Additional File 1.

**Table 3:** GML index for all districts/counties (2009–2018)

Rank	GML	Rank	GML	Rank	GML
1	1.0236	14	0.9891	27	0.9772
2	1.0011	15	0.9872	28	0.9739
3	1.0000	16	0.9870	29	0.9722
4	1.0000	17	0.9868	30	0.9717
5	0.9987	18	0.9867	31	0.9709
6	0.9973	19	0.9865	32	0.9700
7	0.9958	20	0.9860	33	0.9686
8	0.9956	21	0.9856	34	0.9678
9	0.9949	22	0.9824	35	0.9659
10	0.9947	23	0.9810	36	0.9652
11	0.9943	24	0.9810	37	0.9611
12	0.9924	25	0.9786	38	0.9578
13	0.9910	26	0.9772	**Mean**	0.9841

Note: The top-ranked county is County P. All values are presented with four decimal places to ensure precision in the reported data

### Tobit regression results

The correlation exhibited between dependent and explanatory variables is insufficient to cause collinearity issues (coefficient < 0.8). An average VIF (3.450) below 5 indicates the absence of multicollinearity. The Hausman test and the LR test, with *P* > 0.05, support the use of a mixed Tobit model for panel data analysis.

Table 4 shows that the per capita fiscal expenditure (*X*_*2*_) and the urbanisation rate (*X*_*9*_) are positively related to the TFP of PHC institutions at the 1% level. In contrast, the number of nurses per 1,000 residents (*X*_*5*_) is negatively associated with efficiency (at the 5% level), and the number of physicians (*X*_*4*_) shows no significance. Health institution revenue (*X*_*3*_) had a statistically significant positive association with efficiency at the 10% level. The proportion of fiscal expenditure (*X*_*1*_) shows no significant impact. The material inputs like beds per 1,000 residents (*X*_*6*_) and the value of equipment (*X*_*7*_) are also not significant factors. Other variables such as population (*X*_*8*_), per capita GDP (*X*_*10*_) and region (*X*_*11*_) do not have statistically significant effects, either. Validation results of fixed-effects model, random-effects model, mixed Tobit model, and random-effects Tobit model are presented in Table S3, Additional File 1.

**Table 4:** Mixed tobit regression results

Variable	Coefficient	Std. Err.	*P*-value
Proportion of fiscal expenses on PHC institutions (%)	0.0000652	0.0003611	0.857
Per capita fiscal expenses on PHC institutions (CNY)	0.0001962	0.0000644	0. 003***
Total revenue of PHC institutions (CNY)	1.47e-07	8.53e-08	0.085*
Number of physicians per 1,000 resident population	0.0189575	0.0283509	0.504
Number of nurses per 1,000 resident population	-0.0610973	0.0261208	0.020**
Number of beds per 1,000 resident population	-0.0022223	0.0074536	0.766
Value of equipment above 10,000 CNY (10,000 CNY)	3.75e-07	1.67e-06	0.823
Resident population of district/county (10,000 people)	-0.0000964	0.0001801	0.593
Urbanization rate of district/county (%)	0.0006229	0.0002299	0.007***
Per capita GDP of district/county (CNY)	-4.74e-08	1.27e-07	0.708
Region: 1 = Metropolitan circle; 2 = Northeast urban cluster; 3 = Southeast urban cluster	0.0068084	0.0050855	0.182
Constant	0.9181388	0.0282283	< 0.001***

Note: *, **, and *** denote significance at the 10%, 5%, and 1% levels, respectively

## Discussion

### Principal findings

This study revealed a concerning trend of declining efficiency in PHC systems over the past decade, which aligns with findings from various academic studies [6, 40]. This phenomenon can be attributed to multifaceted challenges, including the deteriorating quality of PHC organizations, often plagued by inadequate resources and infrastructural deficits, as well as human resource constraints, particularly the scarcity of skilled healthcare professionals. Moreover, the prevalent public mistrust in grassroots healthcare institutions has led to an increased reliance on larger hospitals, undermining the utilisation of PHC facilities [3, 6, 38]. This situation is compounded by the lack of advanced technological support in PHC settings, which hampers the delivery of efficient and quality care. These systemic issues highlight the urgent need for comprehensive reforms and strategic investments in PHC systems to reverse this downward trend and ensure sustainable healthcare delivery.

According to Tobit regression results, boosting the capacity of county governments to spend allocated budgets is vital for PHC efficiency growth regardless of regional settings. Government and public funding play a pivotal role in PHC efficiency for several reasons: ensuring resource availability, improving manpower and equipment, facilitating access and affordability of PHC services, encouraging public-private collaborations, and establishing strong governance mechanisms. Despite its lagging regional socioeconomics, several factors outside of fiscal input such as system-wide coordination and governance are also key ingredients for PHC efficiency promotion, as demonstrated by past practical experience [34, 41].

The regression analysis revealed a positive association between the total revenue of PHC institutions and their efficiency, albeit at a lower level of statistical significance (10%). This finding suggests that financial resources play a role in the efficiency of PHC institutions, but the impact may be less pronounced or more variable compared to other significant factors. This lower level of significance could be attributed to the considerable variability in revenue data across different PHC institutions or over time, which may attenuate the statistical significance of the relationship. Additionally, other factors may have a stronger influence on efficiency, potentially overshadowing the effect of total revenue. It is important to interpret the relationship between total revenue and efficiency with caution, and further research is needed to better understand this complex relationship.

Counterintuitively, nurse density related negatively to efficiency, albeit this observation is consistent with conclusions drawn from practices in similar developing countries [42, 43]. It may well be attributed to inefficient utilisation of human resources or a suboptimal physician-nurse ratio, where increased input does not yield the expected output, exacerbating challenges in a resource-constrained environment. Moreover, in China, nurses lack prescription authority, limiting the full unleashing of their productivity [44]. Policy tilt should be directed towards: attracting more talents to engage in PHC to catalyse a stepwise optimisation of PHC practitioner structure, compensations like subsidy funds [34] or transferal of excess clinical staff to more efficient PHC centres [45]; refining PHC professional training and giving frequent guidance [34] for service standards upgradation beyond simply adding staff; tying performance evaluation results to efficiency goals for scientific incentives.

Importantly, urbanisation aligns with the national policy direction, and the urbanisation process in the study municipality mirrors the nationwide trend. The increase in the urbanisation rate may foster health system efficiency through the elevation of population density [42] or the reduction of distances to PHC institutions [19]. Thus, for underdeveloped regions aiming to improve PHC efficiency, carefully planned urbanisation initiatives could be leveraged to strategically centralize populations and optimise proximity to care, thereby facilitating the promotion of PHC efficiency.

**Fig. 1:**
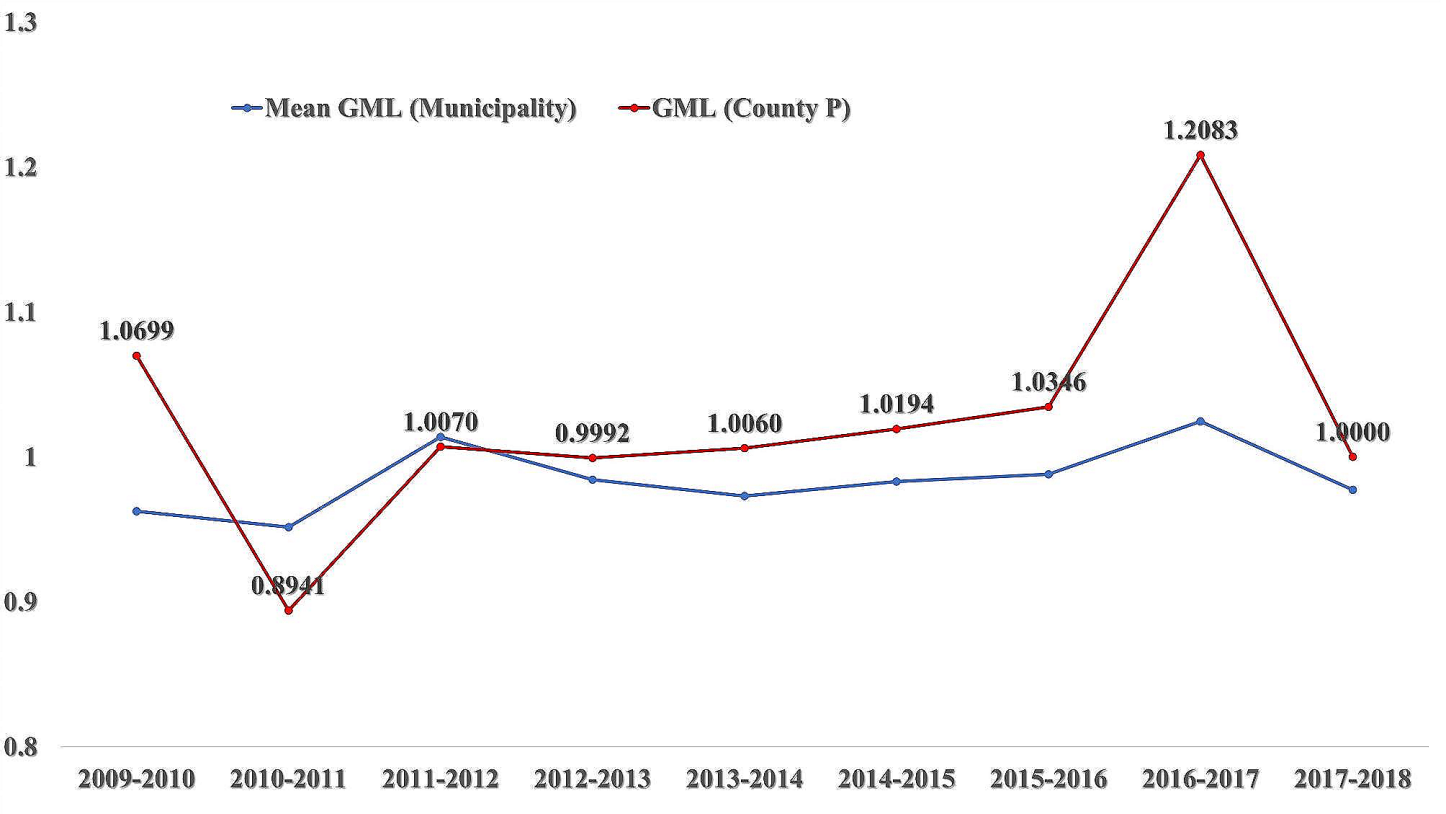
Trends in GML Index Across Years. Note: This figure illustrates the annual changes in the GML index for the entire municipality (blue line) and specifically for County P (red line) from 2009 to 2018. The y-axis represents the GML index, indicating TFP changes, while the x-axis denotes the time

The attainment of the highest efficiency ranking by impoverished County P emerged as an unforeseen finding. To elucidate this finding, a comprehensive literature review was conducted. Previous studies indicating that County P enhanced its PHC capacity and output via horizontal integration and innovative financing reforms were found to corroborate our results [46]. County P implemented gradual integration reform via localised pilots in 2009, with initial effects emerging around 2012 (Fig. 1), and its efficiency has consistently surpassed the municipality-wide average since then. To substantiate the association between the policy impacts of horizontal integration reforms and the efficiency of primary care system reforms, the correlation was re-examined using Tobit modelling (details represented in Table S4, Additional File 1). Tobit regression analysis verifies the horizontal integration reform’s positive association with improved PHC efficiency.

County P’s success is largely attributed to its early adoption of horizontal integration reforms, which involved the consolidation of township and village clinics. This integration facilitated better resource allocation, improved care coordination, and enhanced service delivery at the grassroots level. Furthermore, the integration was complemented by innovative financing reforms, which diversified funding sources and ensured sustained investment in primary care. These reforms not only streamlined PHC services but also increased accessibility for the local population [47].

The study’s Tobit regression analysis further substantiated the positive correlation between these policy reforms and the efficiency of PHC systems. In essence, County P’s experience exemplifies how targeted policy interventions, particularly those fostering integration and financial innovation, can effectively improve the efficiency of PHC organizations. This case serves as a model for other regions seeking to enhance their PHC delivery systems through similar reforms.

### Comparison with existing literature

Our findings are consistent with global trends in healthcare financing and reform, emphasizing the critical role of government investment in achieving efficient and equitable healthcare delivery [48]. Many countries have embarked on integration reforms to align healthcare providers, such as Kaiser Permanente [49], the National Health Service (NHS) model [50], the NHS-type system [51], and informative case studies described by other developed countries [52–54]. Domestically, notable examples include the Sanming model [55] and the Luohu model [56]. In most Chinese counties, dominant county hospitals spearhead hierarchical or vertical integration with PHC providers [8, 57]. While predominantly vertical integration among PHC institutions showed some utility in China [55, 58], our study corroborates past research [46, 59] that reported the effectiveness of horizontal integration reforms in underprivileged County P, potentially complementing mainstream vertical integration efforts.

### Implications of findings

This study indicates that increasing governmental fiscal input and launching horizontal integration reforms may be potential efficiency promoters for PHC. Aligning with the trend of urbanisation may also help attain PHC efficiency gains. However, solely expanding staff volume risks inefficiency, emphasizing the need for efficiency-oriented investment entailing training, incentives, evaluation, and skill mix optimisation to unleash the full potential of the PHC workforce. These findings provide valuable insights for policymakers and healthcare administrators in designing targeted interventions to enhance PHC efficiency, particularly in resource-constrained settings.

### Strengths and weaknesses

Compared to previous efficiency studies, this research has a uniquely long timespan with 10 years of provincial panel data at the county or district level. Some studies leveraged shorter 2-8-year data in other specific provinces [29–31, 34], some were limited to only traditional Chinese medicine hospitals [31], and others revolved around national data at the province level [16, 20, 32]. The application of SBM-DDF-GML methods is distinct from conventional DEA models used in other works [29, 31, 34]. The Tobit regression for influencing factor identification is consistent with some studies [31, 34] but examines different regional cases with particular socioeconomic characteristics. Most studies lacked undesirable outputs, unlike this one. The southwest China municipality innovatively targeted evaluating efficiency in an under-reported province, bearing meaningful research significance.

Specifically, the study helps clarify misconceptions by showing that simply adding staff may not be instrumental in PHC efficiency promotion. Uncovering horizontal integration, strategic financing, and urbanisation as efficiency promoters furnished original evidence to guide reforms. Ultimately, the proposition that horizontal integration may complement mainstream vertical integration in strengthening the PHC system in underprivileged regions in China and beyond leaves room for continued exploration.

Regarding limitations, as PHC facilities shoulder public health responsibilities, this study focused on medical functions with limited data availability on disease control. While emphasizing service volume outputs, quality measures were somewhat overlooked. Additionally, the education factor, an important component of human capital and health-related skills, was not included in the socioeconomic factors of the Tobit regression, which may have provided valuable insights. Generalizability may be limited given the single-province design, nonetheless, the efficiency model and regression results may inform assessments in other regions of China and developing countries with similar PHC challenges. Undoubtedly, this is an associative study instead of rendering definitive causal explanations, leaving open avenues for next-step in-depth investigation.

### Future study

To advance the understanding of PHC efficiency determinants, future research should focus on incorporating additional quality and public health indicators through literature reviews and expert consultations. Furthermore, standardized educational metrics should be integrated to elucidate the role of education in PHC efficiency. Future studies should prioritize the collection and integration of reliable educational data. County P warrants further investigation to uncover more valuable lessons. Large-scale surveys and rigorous qualitative studies to validate causal relationships, discern reform effects, and reveal implementation facilitators, are urgent. Updated longer-term trends data facilitating robust analysis merits further efforts. Future research will extend beyond this current study period to explore the continuing effects of reforms on healthcare efficiency, providing ongoing insights into their evolution and impact. Future studies could examine the impact of different revenue sources on efficiency and explore how PHC institutions allocate and utilise their financial resources. Qualitative research could provide a more nuanced understanding of the financial management practices and challenges faced by PHC institutions in different contexts.

## Conclusions

This study demonstrates horizontal PHC integration, strategic investment prioritization, and proper urbanisation are promising strategies for improving efficiency, while solely expanding staff risks inefficiency without a balanced personnel ratio or skillsets. The enhanced DEA model, GML index, and Tobit regression analysis provide a robust methodological framework that outperforms traditional approaches by effectively handling slack variables, undesirable outputs, and environmental factors. These findings and methods may inform assessments and policy development in other regions facing similar PHC challenges.

The successful experience of County P in implementing horizontal integration reforms and achieving the highest efficiency ranking is particularly noteworthy, corroborating findings from other integration initiatives worldwide. However, the predominance of vertical integration in most Chinese counties underscores the potential for horizontal integration to serve as a complementary approach, especially in underprivileged areas. Further research should validate the causal mechanisms behind County P’s success and explore the applicability of horizontal integration across diverse settings, while also focusing on incorporating additional quality and public health indicators.

Collaboration between policymakers and researchers is essential to translate evidence into practice effectively. By fortifying the knowledge foundation and fostering evidence-informed decision-making, such partnerships can invigorate PHC efficiency and empower these institutions to fulfill their vital role in safeguarding public health, ultimately contributing to the achievement of UHC.

### Electronic supplementary material

Below is the link to the electronic supplementary material.

Supplementary Material 1

## Data Availability

The datasets used and/or analysed during the current study are available from the corresponding author on reasonable request.

## Abbreviations

PHC: Primary Health Care
UHC: Universal Health Coverage
DEA: Data Envelopment Analysis
SFA: Stochastic Frontier Analysis
BCC: Banker-Charnes-Cooper
CCR: Charnes-Cooper-Rhodes
DDF: Directional Distance Function
SBM: Slack-Based Measure
GML: Global Malmquist-Luenberger
PHCPI: Primary Health Care Performance Initiative
TFP: Total Factor Productivity
DMUs: Decision-Making Units
LR: Likelihood Ratio

## References

[1] Croke K, Moshabela M, Kapoor NR, Doubova SV, Garcia-Elorrio E, HaileMariam D (2024). Primary health care in practice: usual source of care and health system performance across 14 countries. Lancet Global Health.

[2] Frieden TR, Lee CT, Lamorde M, Nielsen M, McClelland A, Tangcharoensathien V (2023). The road to achieving epidemic-ready primary health care. Lancet Public Health.

[3] Hanson K, Brikci N, Erlangga D, Alebachew A, De Allegri M, Balabanova D (2022). The Lancet Global Health Commission on financing primary health care: putting people at the centre. Lancet Glob Health.

[4] Zhou J, Peng R, Chang Y, Liu Z, Gao S, Zhao C (2023). Analyzing the efficiency of Chinese primary healthcare institutions using the Malmquist-DEA approach: evidence from urban and rural areas. Front Public Health.

[5] Li X, Lu J, Hu S, Cheng KK, Maeseneer JD, Meng Q (2017). The primary health-care system in China. Lancet.

[6] Li X, Krumholz HM, Yip W, Cheng KK, Maeseneer JD, Meng Q (2020). Quality of primary health care in China: challenges and recommendations. Lancet.

[7] Yip W, Fu H, Chen AT, Zhai T, Jian W, Xu R (2019). 10 years of health-care reform in China: progress and gaps in Universal Health Coverage. Lancet.

[8] Tao W, Zeng Z, Dang H, Lu B, Chuong L, Yue D (2020). Towards universal health coverage: lessons from 10 years of healthcare reform in China. BMJ Global Health.

[9] Qian J, Ramesh M. Strengthening primary health care in China: governance and policy challenges. Health Econ Policy Law. 2023;1–16. 10.1017/S1744133123000257 37846025

[10] Xiong S, Cai C, Jiang W, Ye P, Ma Y, Liu H et al. Primary health care system responses to non-communicable disease prevention and control: a scoping review of national policies in Mainland China since the 2009 health reform. The Lancet Regional Health – Western Pacific [Internet]. 2023 Feb 1 [cited 2023 Dec 18];31. https://www.thelancet.com/journals/lanwpc/article/PIIS2666-6065(22)00009-8/fulltext#%20. 10.1016/j.lanwpc.2022.100390 PMC9985060 36879784

[11] Yip W, Fu H, Jian W, Liu J, Pan J, Xu D (2023). Universal health coverage in China part 1: progress and gaps. Lancet Public Health.

[12] Schwarz D, Duong D, Adam C, Awoonor-Williams JK, Back D, Bang A (2020). Primary care 2030: creating an enabling ecosystem for disruptive primary care models to achieve Universal Health Coverage in Low- and Middle-Income Countries. Ann Glob Health.

[13] Liu X, Wang Z, Zhang H, Meng Q (2021). Measuring and evaluating progress towards Universal Health Coverage in China. J Glob Health.

[14] Hou Y, Tao W, Hou S, Li W (2022). Levels, trends, and determinants of effectiveness on the hierarchical medical system in China: Data envelopment analysis and bootstrapping truncated regression analysis. Front Public Health.

[15] Cantor VJM, Poh KL (2017). Integrated Analysis of Healthcare Efficiency: a systematic review. J Med Syst.

[16] Mei K, Kou R, Bi Y, Liu Y, Huang J, Li W (2023). A study of primary health care service efficiency and its spatial correlation in China. BMC Health Serv Res.

[17] Jacobs R (2001). Alternative methods to examine hospital efficiency: data envelopment analysis and stochastic frontier analysis. Health Care Manag Sci.

[18] Hollingsworth B (2008). The measurement of efficiency and productivity of health care delivery. Health Econ.

[19] Marschall P, Flessa S (2011). Efficiency of primary care in rural Burkina Faso. A two-stage DEA analysis. Health Econ Rev.

[20] Su W, Hou Y, Huang M, Xu J, Du Q, Wang P (2023). Evaluating the efficiency of primary health care institutions in China: an improved three-stage data envelopment analysis approach. BMC Health Serv Res.

[21] Zhang L, Zeng Y, Fang Y (2019). Evaluating the technical efficiency of care among long-term care facilities in Xiamen, China: based on data envelopment analysis and tobit model. BMC Public Health.

[22] Tone K, Tsutsui M, Dynamic DEA (2010). A slacks-based measure approach☆. Omega.

[23] Färe R, Grosskopf S (2010). Directional distance functions and slacks-based measures of efficiency: some clarifications. Eur J Oper Res.

[24] Banker RD, Natarajan R (2008). Evaluating Contextual Variables Affecting Productivity Using Data Envelopment Analysis. Oper Res.

[25] Guo X, Zhang J, Xu Z, Cong X, Zhu Z (2021). The efficiency of provincial government health care expenditure after China’s new health care reform. PLoS ONE.

[26] Oh Dhyun (2010). A global Malmquist-Luenberger productivity index. J Prod Anal.

[27] Chongqing - Wikipedia [Internet]. [cited 2024 May 3]. https://en.wikipedia.org/wiki/Chongqing.

[28] Veillard J, Cowling K, Bitton A, Ratcliffe H, Kimball M, Barkley S (2017). Better Measurement for Performance Improvement in low- and Middle-Income countries: the primary Health Care Performance Initiative (PHCPI) experience of conceptual Framework Development and Indicator Selection. Milbank Q.

[29] Li NN, Wang CH, Ni H, Wang H, Efficiency. and Productivity of County-level Public Hospitals Based on the Data Envelopment Analysis Model and Malmquist Index in Anhui, China. Chinese Medical Journal [Internet]. 2017 Dec 5 [cited 2023 Dec 3];130(23):2836. https://journals.lww.com/cmj/fulltext/2017/12050/efficiency_and_productivity_of_county_level_public.11.aspx. 10.4103/0366-6999.219148 PMC5717863 29176142

[30] Liu Q, Li B, Mohiuddin M. Prediction and Decomposition of Efficiency Differences in Chinese Provincial Community Health Services. International Journal of Environmental Research and Public Health [Internet]. 2018 Oct [cited 2024 Jan 6];15(10):2265. https://www.mdpi.com/1660-4601/15/10/2265. 10.3390/ijerph15102265 PMC6210897 30332771

[31] Zhong K, Chen L, Cheng S, Chen H, Long F (2020). The Efficiency of Primary Health Care Institutions in the counties of Hunan Province, China: data from 2009 to 2017. Int J Environ Res Public Health.

[32] Yan C, Liao H, Ma Y, Wang J (2021). The impact of Health Care Reform since 2009 on the Efficiency of Primary Health Services: a Provincial Panel Data Study in China. Front Public Health.

[33] Yue J, Zhou Y, Ning J, Yin G, Wu Y, Li J (2021). Efficiency and productivity of county-level traditional Chinese medicine hospitals in Hubei Province, China: a retrospective study based on 17 years of panel data. Int J Health Plann Manage.

[34] Cao F, Xi Y, Zheng C, Bai T, Sun Q (2022). How efficient are Basic Public Health Services between Urban and Rural in Shandong Province, China? A Data Envelopment Analysis and Panel Tobit Regression Approach. Risk Manag Healthc Policy.

[35] Kohl S, Schoenfelder J, Fügener A, Brunner JO (2019). The use of Data Envelopment Analysis (DEA) in healthcare with a focus on hospitals. Health Care Manag Sci.

[36] Sun B, Zhang L, Yang W, Zhang J, Luo D, Han C (2016). Data envelopment analysis on evaluating the efficiency of public hospitals in Tianjin, China. Trans Tianjin Univ.

[37] Tobin J (1958). Estimation of relationships for limited dependent variables. Econometrica.

[38] Kontodimopoulos N, Nanos P, Niakas D (2006). Balancing efficiency of health services and equity of access in remote areas in Greece. Health Policy.

[39] Zhao Z, Dong S, Wang J, Jiang Q (2023). Estimating the efficiency of primary health care services and its determinants: evidence from provincial panel data in China. Front Public Health.

[40] Yip W, Fu H, Jian W, Liu J, Pan J, Xu D (2023). Universal health coverage in China part 2: addressing challenges and recommendations. Lancet Public Health.

[41] Barasa E, Musiega A, Hanson K, Nyawira L, Mulwa A, Molyneux S (2021). Level and determinants of county health system technical efficiency in Kenya: two stage data envelopment analysis. Cost Eff Resource Allocation.

[42] Ahmed S, Hasan MZ, MacLennan M, Dorin F, Ahmed MW, Hasan MM (2019). Measuring the efficiency of health systems in Asia: a data envelopment analysis. BMJ Open.

[43] Top M, Konca M, Sapaz B (2020). Technical efficiency of healthcare systems in African countries: an application based on data envelopment analysis. Health Policy Technol.

[44] Muench U, Whaley C, Coffman J, Spetz J (2021). Scope-of-practice for nurse practitioners and adherence to medications for chronic illness in primary care. J Gen Intern Med.

[45] Akazili J, Adjuik M, Jehu-Appiah C, Zere E (2008). Using data envelopment analysis to measure the extent of technical efficiency of public health centres in Ghana. BMC Int Health Hum Rights.

[46] Zeng Z, Tao W, Ding S, Fang J, Wen J, Yao J (2022). Horizontal integration and financing reform of rural primary care in China: a model for low-resource and remote settings. Int J Environ Res Public Health.

[47] Zeng Z, Luo Y, Tao W, Zhang R, Zeng B, Yao J (2024). Improving access to primary health care through financial innovation in rural China: a quasi-experimental synthetic difference-in-differences approach. BMC Prim Care.

[48] Brundtland GH (2022). Public financing for primary health care is the key to universal health coverage and strengthening health security. Lancet Global Health.

[49] Craig DE, Hartka L, Likosky WH, Caplan WM, Litsky P, Smithey J (1999). Implementation of a hospitalist system in a large health maintenance organization: the Kaiser Permanente experience. Ann Intern Med.

[50] Mitchell C, Higgerson J, Tazzyman A, Whittaker W (2023). Primary care services in the English NHS: are they a thorn in the side of integrated care systems? A qualitative analysis. BMC Prim Care.

[51] Cinelli G, Fattore G (2024). The 2022 community-based integrated care reform in Italy: from desiderata to implementation. Health Policy.

[52] Imai Y. Health Care Reform in Japan. 2002.

[53] Nurjono M, Valentijn PP, Bautista MAC, Wei LY, Vrijhoef HJM (2016). A prospective validation study of a Rainbow Model of Integrated Care Measurement Tool in Singapore. Int J Integr Care.

[54] Angus L, Valentijn PP (2018). From micro to macro: assessing implementation of integrated care in Australia. Aust J Prim Health.

[55] Tu WJ, Zhong SF, Liu YK, Zhan J, Liu Q (2019). The Sanming three-in-one model: a potentially useful model for China’s systemic Healthcare Reform. J Am Geriatr Soc.

[56] Wang X, Sun X, Gong F, Huang Y, Chen L, Zhang Y (2018). The Luohu Model: a template for Integrated Urban Healthcare Systems in China. Int J Integr Care.

[57] Tao W, Zeng Z, Dang H, Li P, Chuong L, Yue D (2020). Towards universal health coverage: achievements and challenges of 10 years of healthcare reform in China. BMJ Glob Health.

[58] Wu Q, Xie X, Liu W, Wu Y (2022). Implementation efficiency of the hierarchical diagnosis and treatment system in China: a case study of primary medical and health institutions in Fujian Province. Int J Health Plann Manage.

[59] Zeng Z, Tao W, Liu C, Zhou L, Pan J, Zhang W (2019). Horizontal integration and financial reform of a primary care delivery system in Pengshui County, China: a case study. Lancet.

