# Supplementary material for: Efficiency evaluation and promoter identification of primary health care system in China: an enhanced DEA-Tobit approach
Source: BMC Health Serv Res. 2024 Jul 3;24:777. doi: 10.1186/s12913-024-11244-0 (PMC11223419; doi:10.1186/s12913-024-11244-0)
Supplement: Supplementary file 1 — Supplementary Material 1 [file 12913_2024_11244_MOESM1_ESM.docx]

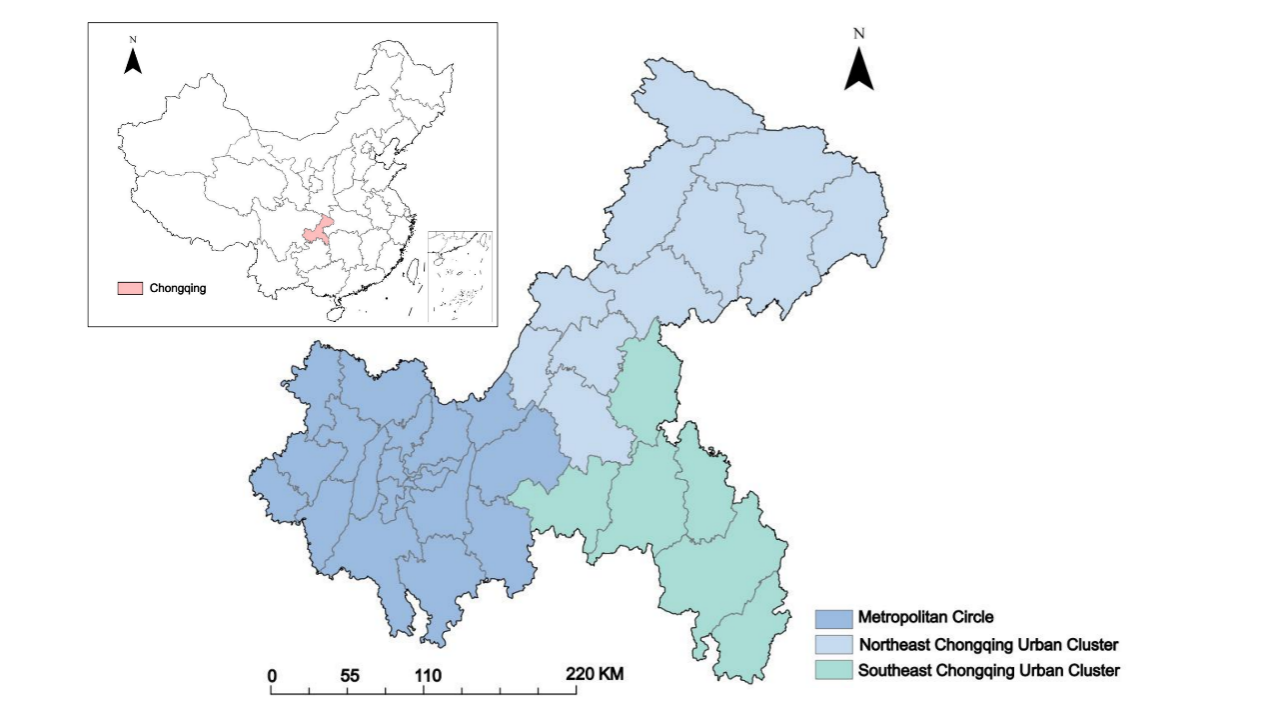


**Figure S1. Map of Chongqing Municipality Showing Regional Divisions**

Note: Geographical representation of Chongqing Municipality within China, illustrating the Metropolitan Circle, Northeast Chongqing Urban Cluster, and Southeast Chongqing Urban Cluster. This map provides a visual delineation of the study area’s urban and rural divisions, highlighting the distinct regions where primary healthcare efficiency was analyzed. The map’s inset shows Chongqing’s location within the national context of China, emphasizing its extensive area and strategic importance.

Table S1. Descriptive statistics of relevant indicators.

| Variable | Number | Mean | Std. Err. | Min | Max |
| --- | --- | --- | --- | --- | --- |
| *Y* | 342 | 0.987 | 0.0718 | 0.716 | 1.392 |
| *X_1_* | 342 | 48.25 | 16.18 | 0.351 | 80.50 |
| *X_2_* | 342 | 118.5 | 57.56 | 0.349 | 287.2 |
| *X_3_* | 342 | 130,197 | 74,806 | 8,781 | 378,300 |
| *X_4_* | 342 | 0.503 | 0.161 | 0.202 | 1.031 |
| *X_5_* | 342 | 0.333 | 0.127 | 0.0855 | 0.660 |
| *X_6_* | 342 | 1.336 | 0.628 | 0.0664 | 2.965 |
| *X_7_* | 342 | 3,714 | 2,828 | 129 | 14,199 |
| *X_8_* | 342 | 78.77 | 33.30 | 18.43 | 166.2 |
| *X_9_* | 342 | 54.70 | 20.46 | 23.80 | 100 |
| *X_10_* | 342 | 43,761 | 25,794 | 8,811 | 182,540 |
| *X_11_* | 342 | 1.605 | 0.746 | 1 | 3 |

**Table S2. GML breakdown (GEC, GTC) for all districts/counties (2009–2018).**

| Rank | GEC | GTC | Rank | GEC | GTC |
| --- | --- | --- | --- | --- | --- |
| 1 | 1.0191 | 1.0045 | 20 | 0.9958 | 0.9902 |
| 2 | 1.0077 | 0.9934 | 21 | 1.0045 | 0.9812 |
| 3 | 1.0000 | 1.0000 | 22 | 0.9993 | 0.9831 |
| 4 | 1.0000 | 1.0000 | 23 | 0.9921 | 0.9888 |
| 5 | 1.0379 | 0.9623 | 24 | 0.9958 | 0.9851 |
| 6 | 1.0120 | 0.9855 | 25 | 1.0000 | 0.9786 |
| 7 | 1.0374 | 0.9599 | 26 | 0.9893 | 0.9878 |
| 8 | 1.0120 | 0.9837 | 27 | 0.9913 | 0.9857 |
| 9 | 1.0113 | 0.9838 | 28 | 1.0000 | 0.9739 |
| 10 | 1.0360 | 0.9601 | 29 | 1.0000 | 0.9722 |
| 11 | 1.0065 | 0.9879 | 30 | 1.0000 | 0.9717 |
| 12 | 1.0236 | 0.9695 | 31 | 1.0000 | 0.9709 |
| 13 | 1.0184 | 0.9730 | 32 | 0.9832 | 0.9867 |
| 14 | 0.9982 | 0.9909 | 33 | 1.0000 | 0.9686 |
| 15 | 1.0042 | 0.9831 | 34 | 1.0000 | 0.9678 |
| 16 | 0.9977 | 0.9893 | 35 | 1.0000 | 0.9659 |
| 17 | 1.0091 | 0.9778 | 36 | 1.0000 | 0.9652 |
| 18 | 0.9974 | 0.9892 | 37 | 0.9759 | 0.9848 |
| 19 | 0.9782 | 1.0085 | 38 | 0.9727 | 0.9847 |

Note: GML=Global Malmquist-Luenberger Index, GEC=General Efficiency Change, GTC=Global Technological Change. All values are presented with four decimal places to ensure precision in the reported data.

To verify the robustness of the regression (11 independent variables) outcomes, the fixed-effects model, random-effects model, mixed Tobit model, and random-effects Tobit model were simultaneously employed for regression analysis. The results are basically consistent.

**Table S3. Regression results of all four models.**

| Variable | Fixed-effects model | Random-effects model | Mixed Tobit model | Random-effects Tobit model |
| --- | --- | --- | --- | --- |
| Proportion of fiscal expenses on PHC institutions (%) | -0.0000242 | 0.0000652 | 0.0000652 | 0.0000652 |
|  | (0.000515) | (0.000367) | (0.000361) | (0.000366) |
| Per capita fiscal expenses on PHC institutions (CNY) | 0.000428** | 0.000196*** | 0.000196*** | 0.000196** |
|  | (0.000161) | (0.0000655) | (0.0000644) | (0.000104) |
| Total revenue of PHC institutions (CNY) | 0.000000344 | 0.000000147 | 0.000000147 | 0.000000147 |
|  | (0.000000222) | (8.67e-08) | (8.53e-08) | (0.000000109) |
| Number of physicians per 1000 resident population | 0.0838 | 0.0190 | 0.0190 | 0.0190 |
|  | (0.0837) | (0.0288) | (0.0284) | (0.0381) |
| Number of nurses per 1000 resident population | -0.204** | -0.0611** | -0.0611** | -0.0611 |
|  | (0.100) | (0.0226) | (0.0261) | (0.0438) |
| Number of beds per 1000 resident population | -0.00994 | -0.00222 | -0.00222 | -0.00222 |
|  | (0.0152) | (0.00758) | (0.00745) | (0.0438) |
| Value of equipment above 10000 CNY (10000 CNY) | -0.00000278 | -0.000000375 | -0.000000375 | -0.000000375 |
|  | (0.00000221) | (0.00000170) | (0.00000167) | (0.00000229) |
| Resident population of district/county (10000 people) | -0.000504 | -0.0000964 | -0.0000964 | -0.0000964 |
|  | (0.00122) | (0.000183) | (0.000180) | (0.000236) |
| Urbanization rate of district/county (%) | 0.00242 | 0.000623** | 0.000623** | 0.000623 |
|  | (0.00177) | (0.000234) | (0.000230) | (0.000512) |
| Per capita GDP of district/county (CNY) | -0.000000712 | -4.74e-08 | -4.74e-08 | -4.74e-08 |
|  | (0.000000398) | (0.000000129) | (0.000000127) | (0.000000297) |
| Region: 1=metropolitan circle; 2=Northeast urban agglomeration; 3=Southeast urban agglomeration | 0 | 0.00681 | 0.00681 | 0.00681 |
|  | (.) | (0.00517) | (0.00509) | (0.00763) |
| constant | 0.880*** | 0.918*** | 0.918*** | 0.918*** |
|  | (0.130) | (0.0287) | (0.0282) | (0.0425) |

*Note:* Significance levels **p < 0.05; ***p < 0.001.

With the addition of the 12th explanatory variable reflecting policy intervention, a positive correlation with PHC efficiency is exhibited in the random-effects model, the mixed Tobit model, and the random-effects Tobit model.

**Table S4. Regression results of all four models.**

|  | Fixed-effects model | Random-effects model | Mixed Tobit model | Random-effects Tobit model |
| --- | --- | --- | --- | --- |
| Horizontal integration reform: 1=implemented; 0=not yet implemented | 0 | 0.0410*** | 0.0410*** | 0.0410*** |
|  | (.) | (0.00206) | (0.00206) | (0.00206) |
| constant | 0.987*** | 0.987*** | 0.987*** | 0.987*** |
|  | (6.24e-11) | (6.24e-11) | (6.24e-11) | (6.24e-11) |
| var(e.tfpch) |  |  | 0.00510*** |  |
|  |  |  | (0.00125) |  |
| sigma_u |  |  |  | 2.18e-22 |
|  |  |  |  | (0.00449) |
| sigma_e |  |  |  | 0.0714*** |
|  |  |  |  | (0.00273) |
| N | 342 | 342 | 342 | 342 |

*Note:* Significance levels **p < 0.05; ***p < 0.001.

**Key Formulas and Equations**

Denote the Kth county as decision making unit (DMU_k_). Assume DMU_k_ employs *N* inputs *x* = ( *x_1_*，…，*x_n_* ) ∈$R_{n}^{+}$ to obtain m expected outputs *y =* ( *y_1_，…，y_n_* ) ∈$R_{m}^{+}$ and *I* undesirable outputs *b =* ( *b_1_，…，b_n_* ) ∈$R_{I}^{+}$. Then the inputs and outputs of DMU_k_ in period *t* can be represented as ( *x^kt^，y^kt^，b^kt^* ) . Based on this, the production possibility set $P^{t}\left( x \right)$ and $P^{G}\left( x \right)$ is constructed, emphasizing consistency and comparability of the production frontier. The production possibility sets of DMU_k_ in period t and the global production frontier are defined below.

$P^{t}(x)=\{\begin{aligned} (y^{t},b^{t}):\sum_{k=1}^{K} z_{k}^{t}y_{km}^{t}\geq y_{km}^{t},\forall m;\sum_{k=1}^{K} z_{k}^{t}b_{ki}^{t}=b_{ki}^{t},\forall i; \\ \sum_{k=1}^{K} z_{k}^{t}x_{kn}^{t}\leq x_{kn}^{t},\forall n;\sum_{k=1}^{K} z_{k}^{t}=1,z_{k}^{t}\geq0,\forall k \end{aligned}\}$ (1)

$P^{G}(x)=\{\begin{aligned} (y^{t},b^{t}):\sum_{t=1k=1}^{T} \sum_{k}^{K} z_{k}^{t}y_{km}^{t}\geq y_{km}^{t},\forall m;\sum_{t=1k=1}^{T} \sum_{k=1}^{K} z_{k}^{t}b_{ki}^{t}=b_{ki}^{t},\forall i; \\ \sum_{i=1}^{T} \sum_{k=1}^{K} z_{k}^{t}x_{kn}^{t}\leq x_{kn}^{t},\forall n;\sum_{t=1}^{T} \sum_{k=1}^{K} z_{k}^{t}=1,z_{k}^{t}\geq0,\forall k \end{aligned}\}$ (2)

Traditional directional distance functions (DDF) can introduce bias when estimating efficiency in the presence of slack variables. Hence, this study redefines the period SBM directional distance function encompassing undesirable outputs.

${\overset{\to}{S}}_{V}^{t}(x^{t,k^{'}},y^{t,k^{'}},b^{t,k^{'}},g^{x},g^{y},g^{b})=\max_{s^{x},s^{y},s^{b}}\frac{\frac{1}{N}\sum_{n=1}^{N} \frac{S_{n}^{x}}{g_{n}^{x}}+\frac{1}{M+I}(\sum_{m=1}^{M} \frac{S_{m}^{y}}{g_{m}^{y}}+\sum_{i=1}^{I} \frac{S_{i}^{b}}{g_{i}^{b}})}{2}$

$\begin{aligned} \text{ s. t. }\sum_{k=1}^{K} z_{k}^{t}x_{kn}^{t}+s_{n}^{x}=x_{k^{'}n}^{t},\forall n;\sum_{k=1}^{K} z_{k}^{t}y_{km}^{t}-s_{m}^{y}=y_{k^{'}m}^{t},\forall m \\ \sum_{k=1}^{K} z_{k}^{t}b_{ki}^{t}+s_{i}^{b}=b_{ki}^{t},\forall i;\sum_{k=1}^{K} z_{k}^{t}=1,z_{k}^{t}\geq0,\forall k;s_{m}^{y}\geq0,\forall m;s_{i}^{b}\geq0,\forall i \end{aligned}$ (3)

Wherein, ($g^{x}$*，*$g^{y}$*，*$g^{b}$) refer to the direction vectors of input reduction, expected output increase and undesirable output decrease, respectively. ($s_{n}^{x}$*，*$s_{m}^{y}$*，*$s_{i}^{b}$) signify the slack vectors, representing the redundant inputs, insufficient expected outputs and excessive undesirable outputs. If greater than 0, the variables indicate the actual inputs and undesirable outputs surpass the frontier, while expected outputs fall below frontier levels. The specific definitions of the period SBM-DDF and global SBM-DDF covering undesirable outputs are:

${\overset{\to}{S}}_{v}^{G}(x^{t,k^{'}},y^{t,k^{'}},b^{t,k^{'}},g^{x},g^{y},g^{b})=\max_{s^{x},s^{y},s^{b}}\frac{\frac{1}{N}\sum_{n=1}^{N} \frac{s_{n}^{x}}{g_{n}^{n}}+\frac{1}{M+I}(\sum_{m=1}^{M} \frac{s_{m}^{y}}{g_{m}^{m}}+\sum_{i=1}^{I} \frac{s_{b}^{b}}{g_{i}^{b}})}{2}$

$\begin{aligned} \text{ s. t. }\sum_{t=1}^{T} \sum_{k=1}^{K} z_{k}^{t}x_{kn}^{t}+s_{n}^{x}=x_{k^{'}t}^{t},\forall n;\sum_{t=1}^{T} \sum_{k=1}^{K} z_{k}^{t}y_{km}^{t}-s_{m}^{y}=y_{km}^{t},\forall m\text{; } \\ \sum_{t=1k=1}^{T} \sum_{k=1}^{K} z_{k}^{t}b_{ki}^{t}+s_{i}^{b}=b_{ki}^{t},\forall i;\sum_{k=1}^{K} z_{k}^{t}=1,z_{k}^{t}\geq0,\forall k;s_{m}^{y}\geq0,\forall m;s_{i}^{b}\geq0,\forall i \end{aligned}$ (4)

By addressing the issues of infeasible solutions and intransitivity commonly occurring in existing indices, a refined Global Malmquist Luenberger (GML) index is proposed. GML represents the change from period *t* to *t+1*. The final SBM–DDF-GML index constructed in this study is presented below.

$GML_{t}^{t+1}=\frac{1+{\overset{\to}{S}}_{V}^{G}(x^{t},y^{t},b^{t};g^{x},g^{y},g^{b})}{1+{\overset{\to}{S}}_{V}^{G}(x^{t+1},y^{t+1},b^{t+1};g^{x},g^{y},g^{b})}=GEC_{t}^{t+1}\cdot GTC_{t}^{t+1}$ (5)

$GEC_{t}^{t+1}=\frac{1+{\overset{\to}{S}}_{V}^{t}(x^{t},y^{t},b^{t};g^{x},g^{y},g^{b})}{1+{\overset{\to}{S}}_{V}^{t+1}(x^{t+1},y^{t+1},b^{t+1};g^{x},g^{y},g^{b})}$ (6)

$\begin{matrix} \mathrm{GTC}_{t}^{t+1}= & \{[1+{\overset{\to}{S}}_{V}^{G}(x^{t},y^{t},b^{t};g^{x},g^{y},g^{b})]/[1+{\overset{\to}{S}}_{V}^{t}(x^{t},y^{t},b^{t};g^{x},g^{y},g^{b})]\}/\{[1+{\overset{\to}{S}}_{V}^{G}(x^{t+1},y^{t+1},b^{t+1} \end{matrix};g^{x},g^{y},g^{b}]/[1+{\overset{\to}{S}}_{V}^{t+1}(x^{t+1!},y^{t+!},b^{t};g^{x},g^{y},g^{b})]\}$ (7)
